# Venous thromboembolism and COVID-19: a single center experience from an academic tertiary referral hospital of Northern Italy

**DOI:** 10.1007/s11739-020-02550-6

**Published:** 2020-11-08

**Authors:** Federica Melazzini, Marta Colaneri, Federica Fumoso, Giulia Freddi, Marco Vincenzo Lenti, Teresa Chiara Pieri, Davide Piloni, Patrizia Noris, Carla Pieresca, Paola Stefania Preti, Mariaconcetta Russo, Angelo Corsico, Guido Tavazzi, Fausto Baldanti, Antonio Triarico, Francesco Mojoli, Raffaele Bruno, Antonio Di Sabatino, on behalf of the San Matteo Pavia COVID-19 Task Force, Nicola Aronico, Gaetano Bergamaschi, Giampiera Bertolino, Silvia Codega, Filippo Costanzo, Roberto Cresci, Angela Delliponti, Giuseppe Derosa, Michele Di Stefano, Francesco Falaschi, Carmine Iadarola, Elisabetta Lovati, Pietro Carlo Lucotti, Alessandra Martignoni, Caterina Mengoli, Emanuela Miceli, Amedeo Mugellini, Chiara Muggia, Elisabetta Pagani, Ilaria Palumbo, Alessandro Pecci, Tiziano Perrone, Carmelo Sgarlata, Luisa Siciliani, Andrea Staniscia, Francesca Torello Vjera, Giovanna Achilli, Andrea Agostinelli, Valentina Antoci, Alessia Ballesio, Francesco Banfi, Chiara Barteselli, Irene Benedetti, Federica Borrelli de Andreis, Michele Brattoli, Francesca Calabretta, Ginevra Cambiè, Roberta Canta, Federico Conca, Luigi Coppola, Elisa Maria Cremonte, Gabriele Croce, Virginia Del Rio, Francesco Di Terlizzi, Maria Giovanna Ferrari, Sara Ferrari, Anna Fiengo, Tommaso Forni, Chiara Frigerio, Alessandra Fusco, Margherita Gabba, Matteo Garolfi, Antonella Gentile, Giulia Gori, Giacomo Grandi, Paolo Grimaldi, Alice Lampugnani, Francesco Lapia, Federica Lepore, Gianluca Lettieri, Jacopo Mambella, Chiara Mercanti, Stefania Merli, Francesco Mordà, Alba Nardone, Luca Pace, Lucia Padovini, Alessandro Parodi, Ivan Pellegrino, Lavinia Pitotti, Margherita Reduzzi, Giovanni Rigano, Giovanni Romito, Giorgio Rotola, Umberto Sabatini, Lucia Salvi, Giovanni Santacroce, Jessica Savioli, Simone Soriano, Carmine Spataro, Debora Stefani, Anna Rita Aliberti, Alessandro Amatu, Laura Anfossi, Eric Arisi, Chiara Baldi, Mirko Belliato, Lorenzo Bellini, Alberto Benzi, Germana Bichisao, Antonia Bolongaro, Andrea Bottazzi, Federica Broglia, Giacomo Bruschi, Luca Caneva, Emanuele Capaccio, Valeria Carboni, Fabrizio Cavalloro, Maria Ciceri, Luca Civardi, Maria Paola Delmonte, Elisa Lucia Domenegati, Federica Ferrari, Fiorenza Ferrari, Marta Ferrari, Marinella Fuardo, Maddalena Margherita Gerletti, Simonetta Gualdana, Marcella Ilardi, Claudia Lo Coco, Giuseppe Maggio, Maria Benedetta Mascia, Simonetta Mencherini, Paola Maria Merati, Silvia Mongodi, Anna Maria Mori, Federica Morgante, Thekla Larissa Niebel, Silvano Noli, Anita Orlando, Michele Pagani, Debora Passador, Simona Pellicori, Luciano Perotti, Raffaella Picchioni, Silvia Poma, Marco Pozzi, Emanuela Preti, Roberta Puce, Danila Katia Radolovich, Gianluca Ragni, Filippo Repossi, Francesca Riccardi, Roberto Rizzardi, Giuseppe Rodi, Emanuela Roldi, Giovanni Romito, Cristina Rossi, Giuseppe Sala Gallini, Fabio Sciutti, Debora Sportiello, Giulia Ticozzelli, Federico Visconti, Silvia Zizzi, Alessandro Bagliani, Corrado Belotti, Chiara Bossi, Andrea Colombo, Costanza Natalia Julia Colombo, Luca Cremascoli, Valentino Dammassa, Roberto Discepoli, Maria Adelaide Garlando, Filippo Grandini, Andrea Pellegrini, Cecilia Quaranta, Andrea Stella, Francesco Torresani, Mario Mondelli, Enrico Brunetti, Angela Di Matteo, Elena Seminari, Laura Maiocchi, Valentina Zuccaro, Layla Pagnucco, Bianca Mariani, Serena Ludovisi, Raffaella Lissandrin, Aldo Parisi, Paolo Sacchi, Savino F. A. Patruno, Giuseppe Michelone, Roberto Gulminetti, Domenico Zanaboni, Stefano Novati, Renato Maserati, Paolo Orsolini, Marco Vecchia, Erika Asperges, Alessandro Di Filippo, Margherita Sambo, Simona Biscarini, Matteo Lupi, Silvia Roda, Ilaria Gallazzi, Michele Sachs, Pietro Valsecchi, Alessandra Ferrari, Bianca Mariani, Matteo Bosio, Alessandro Cascina, Valentina Conio, Rita Di Domenica, Anna Donnetta, Elia Fraolini, Giuseppe Gualtieri, Patrizia Mangiarotti, Francesca Mariani, Federica Meloni, Tiberio Oggionni, Lidia Pasturenzi, Vanessa Ronzoni, Laura Saracino, Giulia Stella, Stefano Tomaselli, Tommaso Abbate, Giulia Accordino, Francesco Bertuccio, Cecilia Burattini, Elisa Cacciatore, Elena Cattaneo, Vittorio Chino, Manuela Coretti, Matteo Della Zoppa, Cristina Infusino, Sara Lettieri, Valeria Maccabruni, Silvia Mancinelli, Claudio Tirelli, Valentina Vertui

**Affiliations:** 1 Department of Internal Medicine, San Matteo Hospital Foundation, University of Pavia, Pavia, Italy; 2 Department of Infectious Disease, San Matteo Hospital Foundation, University of Pavia, Pavia, Italy; 3 Department of Respiratory Disease, San Matteo Hospital Foundation, University of Pavia, Pavia, Italy; 4 Department of Intensive Care, San Matteo Hospital Foundation, University of Pavia, Pavia, Italy; 5 Molecular Virology Unit, Microbiology and Virology Department, San Matteo Hospital Foundation, University of Pavia, Pavia, Italy; 6 Chief Medical Direction, San Matteo Hospital Foundation, Pavia, Italy

**Keywords:** Anticoagulants, Pulmonary embolism, SARS-CoV-2, Thrombosis

## Abstract

Preliminary evidence supports the notion that COVID-19 patients may have an increased susceptibility to develop venous thromboembolism (VTE). However, the magnitude of this association still needs to be defined. Furthermore, clinical predictors of thrombogenesis, and the relationship with the inflammatory status are currently unknown. On this basis, we conducted a retrospective, observational study on 259 consecutive COVID-19 patients admitted to an academic tertiary referral hospital in Northern Italy between March 19th and April 6th, 2020. Records of COVID-19 patients with a definite VTE event were reviewed for demographic information, co-morbidities, risk factors for VTE, laboratory tests, and anticoagulation treatment. Twenty-five cases among 259 COVID-19 patients developed VTE (9.6%), all of them having a Padua score > 4, although being under standard anticoagulation prophylaxis since hospital admission. In the VTE subcohort, we found a significant positive correlation between platelet count (PLT) and either C reactive protein (CRP) (*p* < 0.0001) or lactate dehydrogenase (LDH) (*p* = 0.0013), while a significant inverse correlation was observed between PLT and mean platelet volume (*p* < 0.0001). Platelet-to-lymphocyte ratio significantly correlated with CRP (*p* < 0.0001). The majority of VTE patients was male and younger compared to non-VTE patients (*p* = 0.002 and *p* = 0.005, respectively). No significant difference was found in d-dimer levels between VTE and non VTE patients, while significantly higher levels of LDH (*p* = 0.04) and IL-6 (*p* = 0.04) were observed in VTE patients in comparison to non-VTE patients. In conclusion, our findings showed a quite high prevalence of VTE in COVID-19 patients. Raised inflammatory indexes and increased serum levels of pro-inflammatory cytokines should raise the clinical suspicion of VTE.

**Electronic supplementary material:**

The online version of this article (10.1007/s11739-020-02550-6) contains supplementary material, which is available to authorized users.

## Introduction

Coronavirus disease 2019 (COVID-19) is caused by severe acute respiratory syndrome coronavirus 2 (SARS-CoV-2), which is responsible for acute respiratory distress syndrome in a significant proportion of affected patients (27–31%) [1, 2].

The clinical spectrum of SARS-CoV-2 infection appears to be wide, encompassing asymptomatic infection, mild flu-like syndrome, and severe viral pneumonia with respiratory failure and superimposed bacterial infections which may be fatal [3–5].

Venous thromboembolism (VTE) includes deep vein thrombosis (DVT), pulmonary embolism (PE) and portal vein thrombosis. The association between VTE and COVID-19 was described for the first time by Zhou et al. [6], who identified thrombotic events in 2.9% of a cohort of COVID-19 patients. In previous Asian series, thromboembolic events have been reported in roughly one fourth of COVID-19 patients admitted to the intensive care unit, and these findings correlated with a poor prognosis [7]. A number of pathogenic mechanisms have been hypothesized for VTE in COVID-19 patients, including active inflammation, immobilization and intensive care treatments, but the limited evidence available in the literature does not allow to estimate the relative contribution of each of the above-mentioned factors [8].

Little is known regarding the risk of VTE in non-Asian countries, that are known to have a higher incidence of VTE in comparison to Asian countries [9]. Recent publications reported a high rate of VTE and arterial thrombotic events especially in critically ill patients (10–36% depending on the analyzed cohort), also in Occidental cohorts of COVID-19 subjects [10–15].

Starting from these premises, we here aimed to define VTE rates and types, not considering peripheral and central catheter-related thrombosis, among a cohort of COVID-19 patients during their hospital stay at the San Matteo Hospital Foundation (Pavia, Northern Italy).

## Methods

This is a single-center, retrospective, observational study. We extracted data from medical records of all 259 consecutive patients with a diagnosis of COVID-19 admitted to the Departments of Internal Medicine, Infectious Disease, Intensive Care, and Respiratory Disease of the San Matteo Hospital Foundation (Pavia, Northern Italy), between March 19th and April 6th, 2020. The enrolment was limited to this time lapse as exhaustive data were available only for patients enrolled during this period. All patients had a confirmed diagnosis of COVID-19 by Real Time Polymerase Chain Reaction (RT-PCR) in respiratory samples (see Supplementary materials).

We reviewed records of all 259 COVID-19 patients for demographic information, co-morbidities, risk factors for VTE according to the Padua prediction score [16], laboratory tests and anticoagulation treatment at the time of hospital admission.

DVT and PE were diagnosed through ultrasound and CT-scan or CT-angiography, respectively. In particular, every patient with clinical suspect of VTE or worsening respiratory function, underwent four-points US or CT-scan. The regions tested included: the common femoral vein at the level of inguinal crease, the superficial femoral vein superior to the adductor canal, the popliteal vein in the popliteal fossa and the great saphenous vein. The remaining patients were tested for routine laboratory tests if not daily, at least every 2–3 days. Symptomatic patients were defined as follows: worsening PaO_2_/FiO_2_ despite optimized therapy, signs and symptoms of DVT (i.e., pain, swelling, warmth), increase of d-dimer. No peripheral and central catheter-related DVTs were included in the study.

We also examined the correlation between DVT and selected inflammatory markers, including C-reactive protein (CRP), interleukin (IL)-6, mean platelet volume (MPV), neutrophil-to-lymphocyte ratio (NLR), and platelet-to-lymphocyte ratio (PLR).

The study was performed as a clinical audit using routinely collected clinical data and as such is exempt from the need to require written informed consent. The study was approved by the local ethics committee (San Matteo Hospital Foundation) on March 13th 2020.

The results of this study are reported according to the STrengthening the Reporting of OBservational studies in Epidemiology (STROBE) recommendations [17].

## Statistical analyses

Given the observational nature of the study, sample size was not calculated a priori. Continuous variables were expressed as median and range and compared with the Mann–Whitney *U* test. Categorical variables were described as number (%), and proportions for categorical variables were compared using the Fisher exact test. Spearman correlation coefficient was calculated for relevant laboratory parameters. When variables were not available for some patients, these were excluded for percentage calculation. A two-sided *α* of < 0.05 was considered statistically significant. Statistical analyses were performed using MedCalc (Belgium, version 2020).

## Results

A total of 259 cases of definite COVID-19 patients were included in the registry between March 19th and April 6th 2020. Demographic characteristics, medical history and laboratory parameters of all patients included in the study are reported in Table 1. The median age of the patients was 70 years (range 25–97), 32% of the total were female (median age 66, range 25–94), 68% were male with a median age of 74 (range 27–97).

**Table 1:** Demographic characteristics, medical history and laboratory results of the two whole cohort of COVID-19 patients enrolled in the study, and of the subcohorts of patients with and without VTE at the time of hospital admission

	All patients (*n* = 259)	VTE (*n* = 25)	Non-VTE (*n* = 234)	*p* value
Median age, years (range)	70 (25–97)	62 (44–84)	70 (25–97)	**0**.**005**
Sex				
Female	66 (25–94)	63 (44–84)	69 (25–94)	**0**.**006**
*n*. (%)	(32)	(24)	(32)	
Male	74 (27–97)	62 (51–79)	79 (27–97)	**0**.**002**
*n*. (%)	(68)	(76)	(68)	
Padua score				
> 4	226	25	202	0.4
* n*. (%)	(87)	(100)	(86)	
≤ 4	45	0	45	<** 0**.**0001**
* n*. (%)	(13)	(0)	(14)	
Ventilation				
Non-invasive ventilation	205	16	189	0.2
* n*. (%)	(79)	(64)	(81)	
Invasive mechanical ventilation	54	9	45	0.29
* n*. (%)	(21)	(36)	(19)	
Anticoagulant prophylactic therapy				
Enoxaparin	206	14	192	0.07
*n*. (%)	(79)	(56)	(82)	
Fondaparinux	3	2	1	0.06
* n*. (%)	(1)	(8)	(4)	
Calcic heparin	30	7	23	**0**.**03**
* n*. (%)	(12)	(28)	(10)	
Department				
Subintensive/intermediate care	205	16	189	0.07
*n*. (%)	(79)	(64)	(81)	
Intensive care unit	54	9	45	**0**.**04**
* n*. (%)	(21)	(36)	(19)	
Laboratory parameters				
Haemoglobin (g/dl), median (range)	12.5 (6.8–17.7)	13.4 (8.9–15.5)	12.4 (17.7–6.8)	0.08
Leukocytes (n/µl), median (range)	7.1 (0.2–91.3)	9.2 (3.1–17.7)	6.7 (0.2–91.3)	0.2
Lymphocytes (n/µl), median (range)	0.7 (0.02–86.2)	0.7 (0.2–4.9)	0.7 (0.1–86.2)	0.39
NLR, median (range)	8.2 (0.1–390.5)	13.7 (2.3–25.1)	8.2 (0.1–390.5)	0.5
Platelets (× 10^3^/µl), median (range)	193 (12–768)	184 (80–598)	197 (12–768)	0.7
LDH (mU/ml), median (range)	369 (142–4641)	590 (184–4641)	369 (142–2578)	**0**.**04**
C-reactive protein (mg/dl), median (range)	12.3 (0.2–47.4)	22 (3.5–47.4)	12.2 (0.2–42.5)	**0**.**05**
Procalcitonine (ng/ml), median (range)	0.3 (0–206)	0.4 (0–56.4)	0.3 (0–206)	0.9
d-Dimer (µg/l), median (range)	5181 (555–35,000)	15,350 (3824–35,000)	3229 (555–35,000)	0.09
Interleukin-6 (mg/dl), median (range)	114.9 (5.7–386.4)	163.5 (24.2–386.4)	66.2 (5.7–215.3)	**0**.**04**

Statistically significant p-values are indicated in bold

*LDH* lactate dehydrogenase, *NLR* neutrophil-to-lymphocyte ratio, *PLR* platelet-to-lymphocyte ratio, *VTE* venous thromboembolism

Twenty-five patients turned out to be complicated by VTE (9.6%), of which 21 had DVT, four had PE, and one among them also had portal vein thrombosis. All patients with VTE had a Padua score > 4. We did not identify any incidental diagnosis of VTE, because asymptomatic patients did not undergo diagnostic work-up. Of the 259 enrolled patients, 239 (92.3%) received thromboprophylaxis. Among them, 24 (10.4%) developed VTE.

Twenty-two out of the 25 VTE cohort patients underwent prophylactic therapy with low molecular weight heparin (LWMH) or calcic heparin. Three patients of the VTE-group had clinical evidence of lower DVT by the time of hospital admission, hence anticoagulant therapy was immediately started. The median time lapse between hospital admission and VTE onset was 5.0 days (range 0–5 days). The median time lapse between onset of COVID-19-related symptoms and VTE was 15.0 days (range 10–30 days). DVT was the most frequent event, distributed among upper extremities and lower limb, being upper DVT more prevalent. Of note, the prevalence of VTE in ICU patients was higher in respect to sub intensive/intermediate care patients departments (17% and 8%, respectively; *p* = 0.02). There was a significant correlation between platelet parameters and inflammation. In particular, platelet count directly correlated with either CRP (*p* < 0.0001, *r*_s_ = 0.13392) or LDH (*p* = 0.0013, *r*_s_ = 0.05438), and it inversely correlated with MPV (*p* < 0.0001, *r*_s_ = − 0.75608). MPV significantly correlated in an inverse manner with either CRP (*p* < 0.0001, *r*_s_ = − 0.01657) or LDH (*p* < 0.0001, *r*_s_ = 0.16386). PLR significantly correlated directly with CRP (*p* < 0.0001, *r*_s_ = 0.35769). d-dimer correlated directly with PLT (*p* = 0.006, *r*_s_ = 0.03036), LDH (*p* = 0.007, *r*_s_ = 0.33395) and inversely with MPV (*p* < 0.0001, *r*_s_ = 0.5161) and lymphocyte (*p* < 0.0001, *r*_s_ = − 0.0308). Of note, in the non-VTE group a correlation between PLR and CRP was not observed (*p* = 0.9, *r*_s_ = 0.20984) (Table 2).

**Table 2:** Demographic, clinical and biochemical characteristics of COVID-19 patients at the time of hospital admission

N	Age (years)	Sex	BMI	VTE site	Padua score	Associated diseases	Ventilation	Time span between COVID-19 onset and VTE (days)	Time span between hospital admission and VTE (days)	Anticoagulant prophylactic therapy prescribed at the time of VTE diagnosis (dose)	Ward of admission	PaO_2_/FiO_2_	PLT (× 10^9^/L)	PLR	CRP (mg/dl)	LDH (U/L)	Outcome
1	70	M	33	Upper DVT (Unilateral axillar vein)	5	Asthma, OSAS, DM2	Non mechanical (CPAP)	16	9	Enoxaparin (4000 UI/day)	Internal Medicine	109	104	20.8	6.1	965	Dead
2	50	M	28	Lower DVT (Bilateral femoral vein)	5	Hypertension	Non mechanical (Venturi mask)	15	0	None	Internal Medicine	282	241	301.2	25.2	250	Alive
3	62	M	31	Upper DVT (Bilateral subclavian vein)	6	Hypertension, DM2, ischemic cardiopathy	Non mechanical (CPAP)	11	4	Enoxaparin (4000 UI/day)	Pneumology	266	139	278.0	21.5	599	Alive
4	55	M	26	Upper DVT (Unilateral jugular vein)	6	None	Mechanical	16	6	Calcic heparin (7500 UI × 3/day)	ICU	292	263	461.4	6.2	676	Alive
5	68	M	32	Upper DVT (Unilateral axillar vein)	5	Hypertension, ischemic cardiopathy	Non mechanical (Venturi mask)	10	4	Enoxaparin (4000 UI/day)	Pneumology	250	149	402.7	28.7	576	Alive
6	62	M	31	PE (Bilateral segmental PE)	6	DM2	Non mechanical (CPAP)	10	3	Enoxaparin (4000 UI/day)	Infectious Diseases	182	106	66.2	11.5	334	Alive
7	59	M	30	Upper DVT (Unilateral subclavian vein)	6	Hypertension	Mechanical	15	5	Calcic heparin (7500 UI × 3/day)	Internal Medicine	78	246	492.0	32.4	463	Alive
8	65	M	31	Lower DVT (Unilateral femoral-popliteal axis)	5	None	Non mechanical (Venturi mask)	11	4	Enoxaparin (4000 UI/day)	Infectious Diseases	55	299	498.3	22.4	423	Alive
9	56	M	28	Lower DVT (Unilateral femoral vein)	6	Hypertension	Non mechanical (CPAP)	16	8	Fondaparinux (2.5 mg/day)	Infectious Diseases	294	184	153.3	3.5	381	Alive
10	64	F	35	PE (Unilateral subsegmental PE)	6	Hypertension, obesity	Non mechanical (CPAP)	14	7	Enoxaparin (4000 UI/day)	Infectious Diseases	112	144	204.4	10.0	791	Alive
11	70	M	30	Lower DVT (Unilateral femoral vein)	6	Chronic kidney disease, hypertension	Mechanical	13	3	Calcic heparin (5000 UI × 3/day)	Infectious Diseases	84	105	210.0	37.8	807	Alive
12	70	M	34	Upper DVT (Bilateral axillar vein)	7	Hypertension, obesity, DM2	Non mechanical (Venturi mask)	11	5	Enoxaparin (4000 UI/day)	Pneumology	52	183	183.0	12.1	604	Alive
13	62	F	28	Lower DVT (Unilateral femoral–popliteal axis)	6	None	Non mechanical (Venturi mask)	14	5	Enoxaparin (4000 UI/day)	Infectious Diseases	76	292	7933.6	31.5	812	Alive
14	61	M	32	Lower DVT (Unilateral femoral vein)	6	Hypertension	Non mechanical (CPAP)	25	15	Fondaparinux (2.5 mg/day)	Infectious Diseases	81	249	415.0	9.8	258	Alive
15	44	M	34	Upper DVT (Unilateral axillar vein)	5	None	Mechanical	29	19	Enoxaparin (4000 UI/day)	Internal Medicine	59	503	231.8	42.0	936	Alive
16	79	F	31	Lower DVT (Unilateral femoral–popliteal axis)	8	Hypertension	Non mechanical (CPAP)	16	0	None	Internal Medicine	227	172	860.0	20.2	555	Dead
17	78	M	24	Upper DVT (Unilateral subclavian–axillar axis)	6	Hypertension, dyslipidaemia	Non mechanical (CPAP)	15	8	Enoxaparin (4000 UI/day)	Internal Medicine	65	138	76.7	27.5	472	Alive
18	84	F	24	Lower DVT (Bilateral femoral vein)	6	None	Non mechanical (CPAP)	15	8	Enoxaparin (4000 UI/day)	Internal Medicine	332	100	131.6	18.1	1124	Alive
19	45	M	24	PE (Bilateral subsegmental PE)	5	HBV-related chronic liver disease	Non mechanical (CPAP)	30	20	Enoxaparin (4000 UI/day)	Infectious Diseases	300	598	738.3	44.5	494	Alive
20	66	M	29	Upper DVT (Unilateral subclavian–jugular axis)	6	Dyslipidaemia	Mechanical	13	3	Calcic heparin (5000 UI × 3/day)	ICU	48	146	486.7	24.6	1162	Alive
21	69	M	31	Upper DVT (Unilateral subclavian vein)	6	Hypertension	Mechanical	14	4	Calcic heparin (7500 UI × 3/day)	Internal Medicine	93	244	196.8	47.6	645	Alive
22	48	M	27	Upper and lower DVT (Unilateral axillar vein and unilateral femoral–popliteal axis)	6	None	Mechanical	28	18	Calcic heparin (5000 UI × 3/day)	Infectious Diseases	74	218	311.4	34.9	590	Alive
23	51	F	24	Upper and lower DVT (Unilateral axilla–subclavian axis and unilateral femoral vein)	5	None	Mechanical	16	6	Calcic heparin (5000 UI × 3/day)	Pneumology	112	218	145.3	13.1	4641	Alive
24	59	M	30	Upper DVT (Unilateral subclavian–axillar axis)	6	Hypertension	Mechanical	15	5	Enoxaparin (4000 UI/day)	Internal Medicine	105	304	950.0	40.6	783	Alive
25	64	M	25	PE (Lobar PE) and portal vein thrombosis	5	Rendu Osler disease, hypertension	Non mechanical (Venturi mask)	11	1	Enoxaparin (4000 UI/day)	Internal Medicine	385	80	114.3	24.9	582	Alive

*CPAP* continuous positive airway pressure, *CRP* C-reactive protein, *DM2* diabetes mellitus type 2, *DVT* deep vein thrombosis, *ICU* intensive care unit, *LDH* lactate dehydrogenase, *OSAS* obstructive sleep apnea syndrome, *PaO*_*2*_*/FiO*_*2*_ arterial oxygen partial pressure to fractional inspired oxygen ratio, *PE* pulmonary embolism, *PLR* platelet-to-lymphocyte ratio, *PLT* platelet, *VTE* venous thromboembolism

Furthermore, 76% of VTE patients were male and the median age at admission was 62 (range 44–84), while in the non-VTE group patients were significantly older (median 70, range 25–97), and the male population was less represented. No differences were found between VTE and non-VTE cohorts in terms of anticoagulant prophylactic therapy.

There was no significant difference in d-dimer levels between the VTE and non-VTE patients (*p* = 0.09). LDH was significantly higher in VTE-group compared to the non-VTE group (median 590, range 184–4641 mU/ml). Serum levels of IL-6 were significantly increased in VTE compared to non-VTE patients (*p* = 0.04). All the other parameters did not significantly differ between VTE and non-VTE patients.

Supplementary Table 1 shows characteristics and differences between VTE patients admitted to intensive care unit (ICU) and VTE general ward patients.

## Discussion

Our findings showed a high prevalence (9.6%) of VTE in a non-Asian cohort of COVID-19 patients, which is higher when compared to the prevalence of VTE in patients hospitalized for other causes (1.2–2.0%) [18, 19]. Surprisingly, this high rate of VTE was encountered despite prophylactic anticoagulation therapy in almost all patients. In fact, of all the patients who received thromboprophylaxis, 10.4% developed VTE, which is a higher rate compared to the standard of 0–2% of symptomatic VTE during enoxaparin prophylaxis. In the subcohort of ICU patients, VTE rate was 16.6%, which is rather comparable to the literature-reported rate for these critically ill patients (10–50%, if VTE is screened). Indeed, the VTE rate in our cohort is lower than that from some other recent reports [10, 12]. This might reflect the patients’ setting, since a minority of our patients was managed in the ICU and underwent chest CT-scan.

Surprisingly, 13 out of 25 VTE patients (52%) presented with upper extremities DVT. Of note, 8 of these were ICU patients (62%), while the remaining 5 (38%) were general ward patients and all of them received supportive ventilation through c-PAP. These findings support the notion that intensive care expose critically ill patients to a higher risk of developing DVT. In fact, critically ill patients are at high risk of VTE as they are susceptible to both general risks factors of VTE as well as those specific to ICU patients, such as sedation, immobilization, and use of vasopressors [20]. Furthermore, peripheral lines and peripherally inserted central catheters (PICCs) for intravenous therapy, could have contributed to the thrombotic process, particularly in an inflammatory state like the COVID-19 related one.

The relationship between viral pneumonia and predisposition to VTE has been well described [21, 22]. This phenomenon is the result of interaction between activated leukocytes and cellular adhesion molecules on the vein wall [23–30]. Inflammatory mechanisms up-regulate procoagulant factors, down-regulate natural anticoagulants and inhibit fibrinolytic activity [31]. In particular, as Marongiu et al*.* assert, the massive endothelial damage and activation of coagulation cascade is local [32], leading to pulmonary microvessels thrombosis [33]. Therefore, our study supports the notion that VTE correlates with an inflammatory burst and inflammatory indexes such as LDH, IL-6 and PLR could be useful in stratifying the risk for COVID-19 patients to develop VTE manifestations.

Unfortunately, there is still a gap of knowledge on the certain pathogenesis of VTE related to SARS-CoV-2 infection, so that we cannot exclude that a part of the PE reported were pulmonary thrombosis and not traditional isolated PE [34, 35].

Indexes such as MPV and PLR have been used as unfavorable predictors in other viral infections [36, 37]. The existence of a significant correlation between PLR and inflammatory indexes in the VTE subcohort seems to support the role of this marker as an unfavorable predictor in COVID-19 patients, in keeping with recent reports [38].

In contrast with Tang et al. [39], we did not find any significant difference in d-dimer between the two cohorts and this could be related to the numerical imbalance between VTE and non VTE patients.

Our study also showed that, unexpectedly, platelet count was only slightly reduced or normal in most of COVID-19 patients. That could be explained by the inflammatory state too, since thrombopoietin could increase in response to lung inflammation [40]. Hence, inflammatory profile, more than platelet count and coagulation profile, could help us to determine and estimate the severity of COVID-19. According to recent publications, we believe that enoxaparin and fondaparinux should be preferred instead of calcic heparin because of its anti-inflammatory properties [41]. Moreover, Mycroft-West et al. [42], showed that LMWH can bind to the SARS-CoV-2 surface protein (Spike) S1 receptor binding domain and block the replication of the virus, thus showing potential antiviral effects.

If early initiation of prophylactic anticoagulant therapy should be recommended in COVID-19 patients [8], how to optimize anticoagulant therapy is still a matter of debate [43]. It can be assumed that by implementing the dosage of LMWH traditionally considered as prophylactic, if renal function is permissive and in the absence of bleeding diathesis, most VTE events could be prevented, but further studies are necessary to confirm this therapeutic strategy.

Indeed, this study has some limitations, including the small sample size, the numerical imbalance between VTE and non-VTE cohorts, the lack of follow-up data, and the scarce use of chest CT-scan in the imaging diagnostic protocol of COVID-19 pneumonia at the time of hospital admission. Moreover, even if catheter-related DVTs were not included in the study, we cannot exclude that blood sampling could partially explain such a high incidence.

Nevertheless, our study confirmed the high rate of VTE in COVID-19 patients, especially in those admitted to the ICU. If anticoagulant therapy is then essential in the management of these patients, attention should be paid to the bleeding risk, due to the increased incidence of peptic ulcer disease in COVID-19 patients [44].

The relationship between VTE and inflammation might support the hypothesis that a cytokine burst plays a central role in thrombotic complication in COVID-19 patients. Supporting our speculation, recent works have discussed how hyperinflammation and detrimental immunothrombosis might play a central role in COVID-19 pathophysiology. The short median time lapse between hospital admission and VTE onset suggests that this phenomenon is not correlated with hospitalization, but rather with COVID-19 per se.

Since 100% of VTE patients had a Padua score greater than 4, we suggest performing upper and lower limb venous ultrasound in COVID-19 patients with a score > 4, as well as routinely in ICU patients to identify and treat DVT as soon as possible. We also suggest routine CT scans in patients with hypoxemia that is not fully explained by lung imaging, as well as in those with a profile risk similar to that described in the cohort of VTE patients.

## Electronic supplementary material

Below is the link to the electronic supplementary material.

Supplementary file1 (DOCX 20 KB)
