# Supplementary material for: Venous thromboembolism and COVID-19: a single center experience from an academic tertiary referral hospital of Northern Italy
Source: Intern Emerg Med. 2020 Nov 8;16(5):1141–52. doi: 10.1007/s11739-020-02550-6 (PMC7648897; doi:10.1007/s11739-020-02550-6)
Supplement: Supplementary file 1 — Supplementary file1 (DOCX 20 KB) [file 11739_2020_2550_MOESM1_ESM.docx]

**Supplementary Materials**

**Methods**

*Real Time Polymerase Chain Reaction*

Total nucleic acids (DNA/RNA) were extracted from 200 µl of samples using the QIAsymphony® instrument with QIAsymphony® DSP Virus/Pathogen Midi Kit (Complex 400 protocol) according to the manufacturer’s instructions (QIAGEN, Qiagen, Hilden, Germany). Specific real-time RT-PCR targeting RNA-dependent RNA polymerase and E genes were used to detect the presence of SARS-CoV-2 according to the WHO guidelines [1] and Corman et al. protocols [2].

**Supplementary Table 1.** Laboratory results of the two ICU and non-ICU patients subcohorts with VTE at the time of admission

|  | ICU patients  (n=9) | Non-ICU patients  (n=16) | P value |
| --- | --- | --- | --- |
| Sex  F/M (%) | 12/88 | 25/75 | **0.002** |
| Age | 58 (44-70) | 64 (45-84) | **0.005** |
| Hb (g/dl), median (range) | 12.7 (8.9-15.5) | 13.4 (10.8-15.4) | 0.3361 |
| Leukocytes (n/ul), median (range) | 10.5 (4.8-15.5) | 6.8 (3.1-17.7) | **0.0576** |
| Lymphocytes (n/ul), median (range) | 0.57 (0.3-2.2) | 0.78 (0.2-4.8) | 0.612 |
| NLR, median (range) | 17.47 (3-25.14) | 6.6 (2.2-23.4) | **0.0016** |
| PLR, median (range) | 311.4 (120.2-950) | 241.2 (20.9-860) | 0.5093 |
| LDH (mU/ml), median (range) | 783 (463-4641) | 483 (184-1124) | 0.3795 |
| CRP (mg/dl), median (range) | 32.5 (6.2-47.6) | 20.9 (3.5-44.5) | **0.0276** |
| PCTI (ng/ml), median (range) | 0.7 (0-56.4) | 0.3 (0.1-2.2) | 0.9407 |
| D-dimer (ug/l), median (range) | 15350 (3824-35000) | 20250 (5500-35000) | 0.5471 |
| IL-6 (mg/dl), median (range) | 160.2 (24.2-254.9) | 274.9 (163.5-386.4) | **0.0556** |

CRP=C reactive protein; F/M=female/male; Hb=hemoglobin; ICU= intensive care unit; IL=interleukin; LDH= lactate dehydrogenase; NLR= neutrophil-to-lymphocyte ratio; PCTI=procalcitonine; PLR= platelet-to-lymphocyte ratio; VTE=venous thromboembolism

**References**

1. World Health Organization. Diagnostic detection of 2019-nCoV by real-time RT-PCR. Version 2, valid from 2020-01-17. https://www.who.int/docs/default-source/coronaviruse/protocol-v2-1.pdf?sfvrsn=a9ef618c_2. Last visited 2020 Jan 19
2. Corman VM, Landt O, Kaiser M, et al (2020) Detection of 2019 novel coronavirus (2019-nCoV) by real-time RT-PCR. Euro Surveill 25:2000045
